# Effects of irrigation and fertilization management on kiwifruit yield, water use efficiency and quality in China: A meta-analysis

**DOI:** 10.3389/fpls.2025.1534702

**Published:** 2025-10-15

**Authors:** Yuehua Ding, Chunfeng Shang, Long Zhao, Shanshan Jin, Chenyang Li, Shanshan Yin, Golam Jalal Ahammed

**Affiliations:** ^1^ College of Horticulture and Plant Protection, Henan University of Science and Technology, Luoyang, China; ^2^ College of Agricultural Equipment Engineering, Henan University of Science and Technology, Luoyang, China; ^3^ College of Information Engineering, Henan University of Science and Technology, Luoyang, China

**Keywords:** meta-analysis, *Actinidia chinensis*, fertilizer management, irrigation, fruit quality, yield, WUE

## Abstract

The water-saving and fertilizer-reduction strategies is important for sustainable agricultural development. However existing kiwifruit water-saving and fertilizer-reduction studies showed significant contradictions in the results of water and fertilizer management. Studies have reported conflicting findings on irrigation: some suggest over-irrigation increases yield and WUE, while others advocate low-volume irrigation. Consequently, this study offers a comprehensive meta-analysis encompassing 1038 observations, with the objective of evaluating the influence of water management and optimized fertilization on the yield, water use efficiency (WUE), and quality of kiwifruit. The results showed that the response of kiwifruit to water management was particularly significant in areas with annual average rainfall > 800 mm and field water holding capacity > 28%, and excessive irrigation had a greater negative impact on yield and WUE. With the increase of tree age, the yield-increasing effect of kiwifruit on water and fertilizer optimization gradually weakened. In terms of irrigation methods, drip irrigation has more advantages than traditional irrigation methods. Reducing super-optimal input (SOI) water input can increase kiwifruit yield by 16.24% and WUE by 20.06%. In terms of fertilization management, reducing the input of SOI nitrogen fertilizer can significantly increase the yield of kiwifruit by 32.76%, while reducing the input of SOI nitrogen, phosphorus and potassium can increase the yield by 3.45%. The contents of soluble sugar and vitamin C increased by 6.35% and 18.37%, respectively, but the contents of titratable acid and soluble solids decreased by 4.35% and 6.18%, respectively. In addition, the optimal nitrogen fertilizer level for kiwifruit varies from region to region, and it is generally recommended to be between 100 – 105 kg/ha per hectare. In summary, scientific and reasonable water and fertilizer management can significantly improve the yield and quality of kiwifruit, optimize WUE, and reduce water and fertilizer waste, providing theoretical basis and practical guidance for sustainable agricultural development.

## 1 Introduction

*Kiwifruit*(Actinidia chinensis), renowned for its high nutritional content, is a widely cultivated fruit abundant in vitamin C, carbohydrates, proteins, and essential minerals (Zhao et al., 2023; Wu et al., 2023; Satpal et al., 2021). A marked increase in global kiwifruit production has been observed, rising from 3.622 million tons in 2014 to 4.518 million tons in 2020. Concurrently, the cultivation area expanded from 224,000 hectares to 280,000 hectares over the same period (Li, 2022). In China, kiwifruit production is predominantly concentrated in the central, southern, and southwestern regions, where both the planting area and total yield surpass those of other regions; however, the yield per unit area remains relatively modest (Lu et al., 2016).This phenomenon shows that while expanding the planting area helps to increase total yield, optimizing water and fertilizer management is urgently needed to improve yield per unit area.Water and fertilizer serve as foundational components of agricultural production, with fertilizer constituting approximately one-third of the overall management expenses in kiwifruit orchards. The prevalent practice of excessive fertilization and irrigation in China, aimed at mitigating the risk of yield loss, has led to significant resource wastage and environmental degradation, including the eutrophication of groundwater and leaching of soil nutrients (Wang et al., 2018a, 2018b; Lv et al., 2020; Cui and Shoemaker, 2018; Li et al., 2017; Davidson et al., 2015). As societal awareness of ecological issues and the economic implications of chemical fertilizers grows, the adoption of fertilizer- and water-saving strategies in agriculture has gained considerable momentum. Water and fertilizer serve as foundational components of agricultural production … the adoption of fertilizer- and water-saving strategies in agriculture has gained considerable momentum (Wang et al., 2019).

A study suggested that optimizing irrigation schedules based on soil water balance is an effective approach, particularly in arid regions, providing a solution for efficient water management in flood-irrigated maize fields (Yi et al., 2022).

Efficient use of water and fertilizer is critical for sustainable agricultural development, especially in arid and semi-arid regions where resource scarcity and environmental concerns are intensifying. The term super-optimal inputs refers to agricultural practices that exceed the optimal levels of water and nutrient application, often leading to resource wastage, reduced efficiency, and environmental degradation. In contrast, water-saving strategies aim to maximize crop productivity per unit of water applied, through techniques such as deficit irrigation or improved irrigation scheduling. Similarly, fertilizer-reduction refers to strategies that maintain yield while decreasing fertilizer input, often by optimizing nutrient timing, type, and dosage through data-driven approaches (Wu et al., 2025; Yi et al., 2022).

However, existing studies on irrigation and fertilization often show inconsistent results, particularly across different climates, cropping systems, and management scales. Many have focused on localized experiments with limited transferability to broader policy or regional planning. Gong et al. (2025) proposed a dynamic optimization of soil phosphorus status (DOP) approach that reduces phosphorus use by up to 47.4% in China without sacrificing yield, offering a model of nutrient management that integrates spatial heterogeneity, crop-specific requirements, and long-term planning (Gong et al., 2025).

These findings highlight the urgent need to integrate more regionally adaptive and resource-efficient management practices into irrigation scheduling and fertilization planning. In particular, flood-irrigated maize fields with varying cultivation histories require context-sensitive strategies to balance productivity with environmental sustainability.

.The previous section discussed the relationship between kiwifruit yield and water and fertilizer management, especially the effect of fertilization on yield. Plant nutrition plays a pivotal role in crop management, with nitrogen being an indispensable nutrient for both crop growth and agricultural yields (Kuypers et al., 2018; Wang et al., 2021; Yang et al., 2017; Tilman et al., 2011). The strategic application of nitrogen fertilizer is widely recognized as a key practice for enhancing crop productivity. However, it is crucial to note that the relationship between crop yield and nitrogen fertilizer input is not linear (Li Y. et al., 2019; Li M.F. et al., 2019; Gu et al., 2017; Hawkesford, 2014). As the amount of fertilizer gradually increases, reaching an optimal level, continuous fertilization beyond this point can lead to a decrease in yield. Studies have indicated that excessive nitrogen application rates, surpassing 50 kg/ha, can adversely affect kiwifruit yield (Pinto et al., 2021). Over-application of nitrogen can result in the substantial accumulation of nitrate nitrogen in the soil profile, thereby altering the soil’s physical and chemical properties (Edwards et al., 2018; Zhou et al., 2016). Nutrient elements are adjusted throughout the growth cycle of kiwifruit according to its growth needs and environmental conditions to ensure its healthy growth and improve yield and quality.

Furthermore, the balanced input of the primary nutrients—nitrogen, phosphorus, and potassium—is essential not only for manipulating yield but also for influencing the quality of kiwifruit. Optimal fertilizer application has been shown to yield the highest returns, with the application of nitrogen and potassium having minimal impact on fruit acidity and soluble solids, although it can compromise fruit firmness (Pacheco et al., 2008). The role of nitrogen and phosphorus in sustaining the yield and quality of ‘Hayward’ kiwifruit has been highlighted (Guarçoni and Ventura, 2011). The differential demands of kiwifruit for these nutrients suggest a propensity for efficient phosphorus utilization, with potentially lower soil phosphorus requirements and higher soil potassium demands (Liu et al., 2000). The impact of nitrogen and potassium application rates, ranging from 125 to 250 kg/ha for nitrogen and a fixed 200 kg/ha for potassium, on the mineral composition of kiwifruit has been significant (Santoni et al., 2016). Despite the existing body of research on the individual effects of nitrogen, phosphorus, and potassium on kiwifruit yield and quality, a comprehensive analysis of the combined application of these fertilizers and the implications of reducing their application rates on kiwifruit yield and quality is lacking. The optimal input (OI) levels of nitrogen (N), phosphorus (P) and potassium (K) in kiwifruit planting are determined according to the specific soil, climate and planting varieties in different regions. For example, the optimal nitrogen, phosphorus and potassium levels of Wuzhi No.3 kiwifruit planted in Heping County, Guangdong Province are: nitrogen (N) 100 – 105 kg/ha, phosphorus (P) 135 – 140 kg/ha, potassium (K) 170 – 175 kg/ha. These values can be regarded as the critical values of nitrogen, phosphorus and potassium in kiwifruit planting under certain conditions. It should be noted that these values are not fixed, but need to be adjusted according to specific soil test results, local climatic conditions and rootstock varieties. If the amount of fertilizer is insufficient, it can be adjusted by increasing the amount and frequency of fertilizer application; however, excessive fertilization should be avoided. If too much fertilizer is applied, the fertilizer concentration in the soil can be diluted by a large amount of water, and measures to remove damaged leaves and prune damaged roots can be taken. Therefore, a quantitative and systematic synthesis of how varying fertilization levels affect kiwifruit yield and quality is imperative for informed practical application.

Furthermore, research has shown that optimizing phosphorus fertilizer application can lead to a substantial reduction in phosphorus use. A dynamic optimization approach demonstrated a 47.4% reduction in phosphorus fertilizer use without any adverse effects on yield, offering a more sustainable solution for agricultural fertilizer management in China (Gong et al., 2025).

Water serves as a fundamental resource for agricultural productivity, with irrigation being a significant consumer of freshwater resources (Busschaert et al., 2022). In China, agriculture, particularly irrigation, is the primary user of water, and the scarcity of water resources poses a notable constraint on the agricultural sector’s development (Li et al., 2020; Li et al., 2021). Research indicates that judicious application of deficit irrigation can often optimize crop water productivity (Wu et al., 2021; Khapte et al., 2019; Patanè and Cosentino, 2009). Kiwifruit, recognized for its substantial water requirements among deciduous fruit trees, necessitates the optimization of irrigation practices to curtail water usage while sustaining or enhancing yield (Gao et al., 2023). Observations reveal that within a specific range, an increase in irrigation volume correlates with elevated levels of reducing sugars and vitamin C in kiwifruit. However, beyond this threshold, a continued increase in irrigation can lead to a marked reduction in titratable acidity, soluble solids, and dry matter content (Wang et al., 2018a; Wang et al., 2018b). Additionally, excessive irrigation combined with high fertilization rates can exacerbate nutrient leaching, thereby impacting crop yields (Muhammad et al., 2022; Wang et al., 2021). Consequently, integrating irrigation and fertilization management at the field scale is deemed critical for the sustainability of agricultural systems (Dai et al., 2019).

However, there are some contradictions in the existing research on the effects of irrigation and fertilizer application. The existing research on water saving and weight loss of kiwifruit shows that there is a significant contradiction between water saving and fertilization management results. In light of this, a meta-analysis is warranted to synthesize the overarching findings from a broad spectrum of existing research (Hedges et al., 1999). The utility of meta-analysis lies in its capacity to account for the diverse water and fertilizer use efficiencies reported in various studies, and to provide a comprehensive examination of the effects of water and fertilizer optimization on kiwifruit trees, an area that has been previously understudied. It allows for the systematic dissection of a multitude of potential factors that could influence dependent variables, thereby enabling the extraction of meaningful conclusions from a body of literature (Stanley, 2001). Consequently, the present study undertakes a meta-analytic review of field trials related to kiwifruit production, with the following three objectives: (1) To evaluate the effects of fertilization and irrigation on kiwifruit yield in China, considering factors such as average annual rainfall, soil water retention capacity and irrigation techniques; (2) To analyze the effects of different irrigation and fertilization practices on water use efficiency (WUE) of kiwifruit; (3) To assess the effects of nitrogen, phosphorus and potassium fertilizers on the quality indexes of kiwifruit.

## 2 Materials and methods

### 2.1 Data acquisition

We searched the Web of Science, CNKI, Wanfang and Google Scholar databases for research literature published from May 2002 to February 2023 on the effects of irrigation and fertilization management on kiwifruit yield, WUE and quality. Our search strategy includes keywords such as ‘ kiwifruit ‘, ‘ fertilization ‘, ‘ irrigation ‘, ‘ nutrient ‘, ‘ yield ‘, ‘ WUE ‘ and ‘ quality ‘, which are not limited by publication date or language. In order to reduce the risk of data bias and ensure the reliability of the study, we have developed specific criteria for the selection of papers:

1. We included studies based on field trials, excluding those conducted in greenhouses or with potted plants.
2. Studies were required to present kiwifruit yield data, with water use efficiency(kg/m^3^) defined as the ratio of yield to water consumption at the field level (WUE = yield/water consumption), which is also known as WUE.
3. We prioritized studies with a randomized block experimental design, favoring those with data repeated over at least two years. Studies lacking repetitive data were excluded. Observations from different years and locations within a single study were treated as independent experiments and included in our dataset.
4. The studies must have reported or allowed the calculation of specific nitrogen, phosphorus, and potassium fertilizer applications.
5. For all variables, studies had to present the mean, sample size (n), standard error (SE), or standard deviation (SD), either in tabular or graphical form. If only n and SE were provided, SD was calculated as SD = SE $\sqrt{n}$ . In cases where SE and SD were not reported, SD was estimated as the mean value multiplied by 0.1. Data presented graphically were extracted using the WebPlotDigitizer-4.2.
6. We excluded data about the interaction between water and fertilization effects; the included data were categorized into water or fertilization subsets.

**Figure 1:**
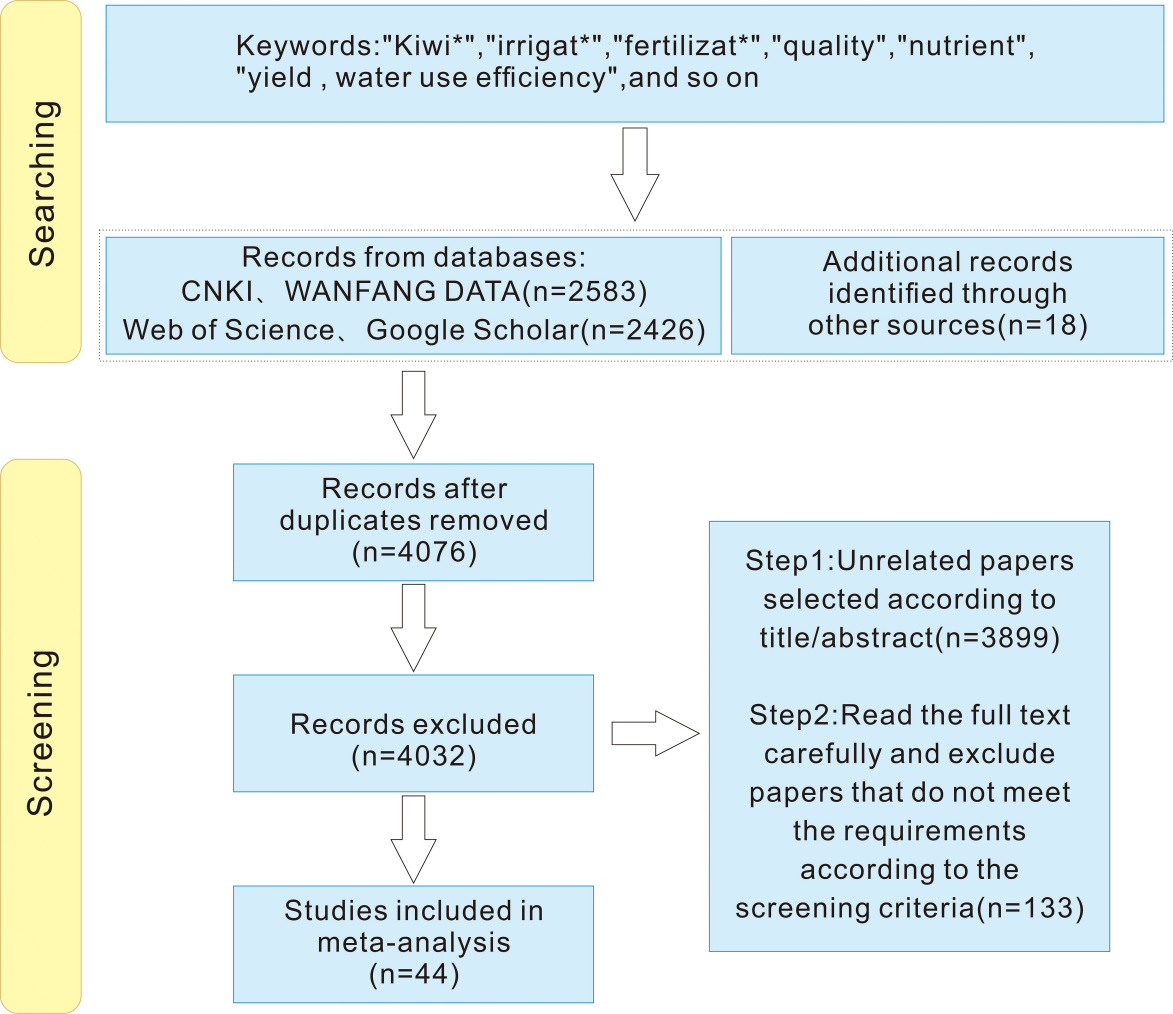
Database literature retrieval screening flow chart.

**Figure 2:**
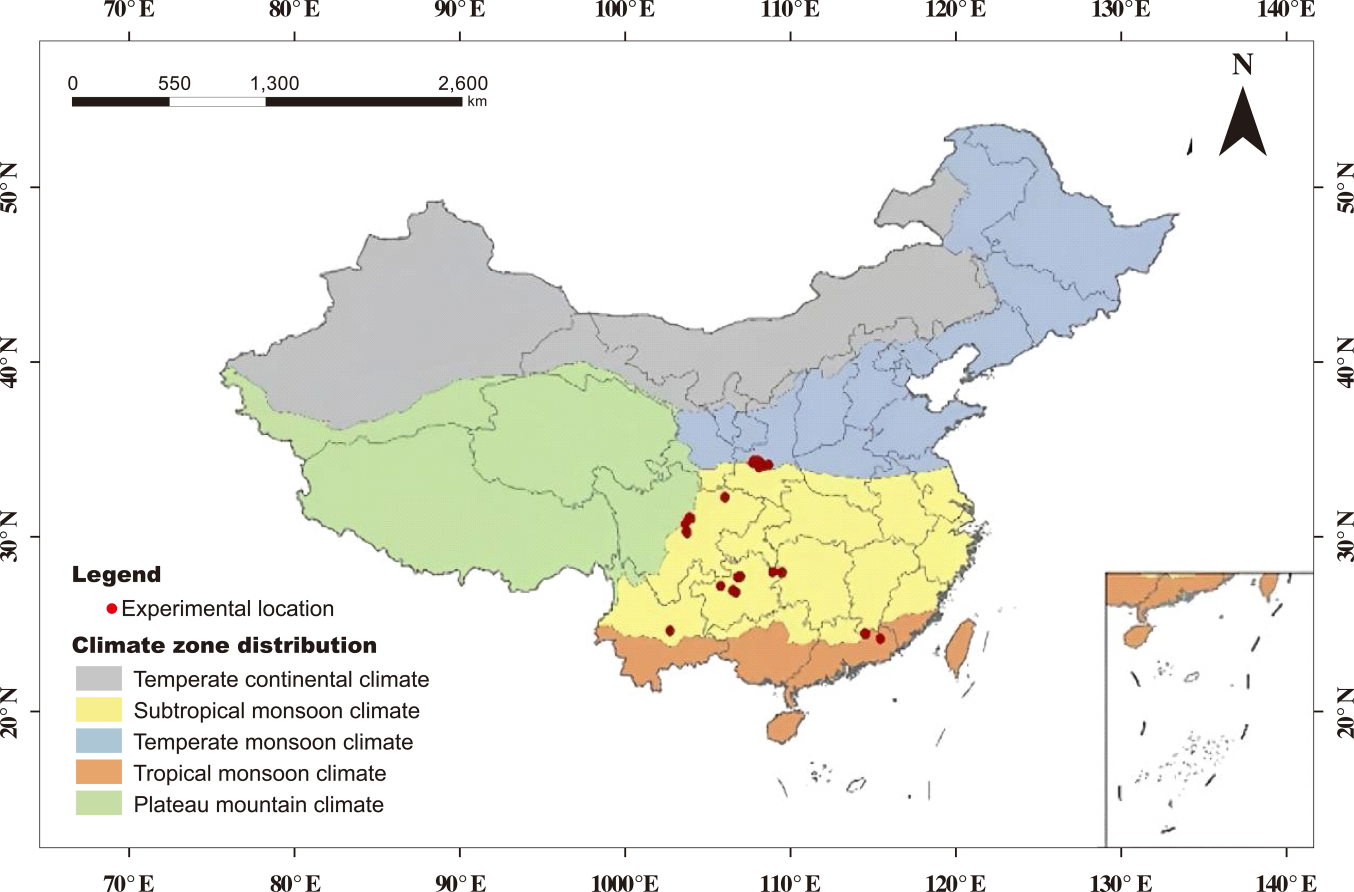
Field trial sites in China included in this meta-analysis.

The literature screening process is depicted in **Figure 1**. Additional data not readily available in publications, such as annual average rainfall, were sourced from the National Meteorological Science Data Center. Considering the varying climatic, edaphic, and irrigation conditions in China’s kiwifruit cultivation areas, we categorized the annual average rainfall into ≤ 800 mm and > 800 mm, the field water holding capacity into ≤ 28% and > 28%, and tree age into ≤ 8 years and > 8 years. These threshold values for annual rainfall (>800 mm) and field capacity (>28%) were determined based on the mean and interquartile range of the collected dataset. These cutoffs correspond to hydrological conditions where excessive water input can increase risks of nutrient leaching and root zone hypoxia, particularly in humid areas (Gao et al., 2023; Wang et al., 2018b). Irrigation systems were classified as drip irrigation and other systems, including small pipe outflow, micro-sprinkler irrigation, and combined drip and sprinkler systems. Ultimately, we amassed data from 44 publications comprising 1038 paired observations, which were categorized into 328 observations for kiwifruit yield, 624 for fruit quality, and 86 for WUE (kg/m^3^) The geographical distribution of the experimental sites is illustrated in **Figure 2**. Details of the publications and data lists are given in **Supplementary Materials**.

### 2.2 Subgroup analysis

The term “ OI of water and fertilizer” denotes the minimum amount required to achieve maximum yield. The optimum levels of nitrogen, phosphorus and potassium in kiwifruit cultivation should be determined according to local specific soil, climate and planting varieties, such as Wuzhi No.3 kiwifruit planted in Heping County, Guangdong Province. The optimum levels of nitrogen, phosphorus and potassium were N: 100 - 105kg/ha, P: 135 - 140kg/ha, K: 170 - 175kg/ha. The absence of fertilization is indicated as “None.” The input of nitrogen fertilizer is categorized as “N” when it exceeds the sub-optimal input (SBI) level (N+) but is less than the SOI level (N-), with the inputs of phosphorus and potassium fertilizers held constant. Similarly, “P” and “K” represent the inputs of phosphorus and potassium fertilizers, respectively, which are above the sub-optimal (P+, K+) but below the SOI (P−, K−) levels. The combination “NK” signifies a scenario where the input of potassium fertilizer is below the optimal level, while the nitrogen fertilizer input is above (N+K-) but below (N-K-) the optimal nitrogen input. Conversely, “PK” indicates that with the potassium fertilizer input below the optimal level, the phosphorus fertilizer input is above (P+K-) but below (P-K-) the optimal phosphorus input. The acronym “NPK” refers to simultaneous changes in the ratios of nitrogen, phosphorus, and potassium fertilizers. It encompasses four combinations: N+P+K+, N+P+K-, N-P-K+, and N-P-K-. Here, N+P+K+ and N+P+K- imply nitrogen and phosphorus fertilizer inputs are above the optimal levels when the potassium input is either above or below the optimal level. N-P-K+ and N-P-K- indicate nitrogen and phosphorus fertilizer inputs are below the optimal levels when the potassium input is above or below the optimal, respectively. If the fertilizer input is insufficient, the amount and frequency of fertilization can be increased, but at the same time, too much fertilization should be avoided. If the fertilizer input is too much, the fertilizer concentration in the soil can be diluted by a large amount of water to remove the damaged leaves and trim the damaged roots. In each study included in the meta-analysis, the amount of fertilizer was adjusted according to the nutrient level in the soil. In the **Supplementary Material**, the data on nitrogen, phosphorus and potassium levels in the soil of the test site were supplemented. High water level (W+) and low water level (W−) are defined relative to the optimal water input, with high indicating above and low indicating below this benchmark. High water level (W+) and low water level (W−) are defined as water input higher and lower than the optimal water input, respectively. The optimum water level is the basis of water adjustment, which is determined by the growth stage, climatic conditions, soil type, soil moisture and root distribution range of kiwifruit. Inputs above and below these levels reduce yield and WUE. We compared the observed yield and maximum yield of each study to assess how much yield and WUE were reduced by non-optimal inputs. When performing subgroup analysis, if the number of subgroup data is less than two, the subgroup is not considered.For clarity, the following abbreviations were used throughout the figures and analysis: W+ (super-optimal water input), W− (sub-optimal water input), N+ (nitrogen input above optimal level), N− (nitrogen input below optimal level), and similarly for P+/P− and K+/K−.

### 2.3 Meta-analysis

To quantify the effects of non-optimal water and fertilizer inputs on different response variables (kiwifruit yield, quality, and WUE), the natural logarithm of the response ratio (RR) was used as a measure of the effect size in this meta-analysis, and the ratio of the given variable ( $X_{obs}$ ) in the treatment group to the given variable ( $X_{\max}$ ) in the control group was used to calculate RR as follows:

Effect sizes were computed as log response ratios (Equation 1); the sampling variance (Equation 2) and study weights (Equation 3) were derived accordingly. The overall mean effect (Equation 4) and its 95% confidence interval (Equation 5) were estimated under a random-effects model, and heterogeneity was assessed using I^2^ (Equation 6).


(1) $$RR=\ln(\frac{X_{obs}}{X_{\max}})=\ln(X_{obs})-\ln(X_{\max})$$

Among them, the average yield, quality, and WUE of kiwifruit under non-optimal water and fertilizer input are the quality and WUE values related to the optimal water and fertilizer input (the highest yield).

The variance (*v)* of the effect value can be calculated based on sample size, mean, and standard deviation (Luo et al., 2006):


(2) $$v=\frac{S_{obs}^{2}}{n_{obs}X_{obs}^{2}}+\frac{S_{\max}^{2}}{n_{\max}X_{\max}^{2}}$$

Where, $S_{obs}$ , $n_{obs}$ ,and $X_{obs}$ ,are the standard deviation, sample size and mean of the non-optimal input (treatment group), and $S_{\max}$ , $n_{\max}$ , $X_{\max}$ ,are the standard deviation, sample size and mean of the OI (control group), respectively. Due to the different statistical accuracy of the data in each study, the weighted response ratio (RR + +) was used to calculate the effect value of the treatment group and the control group to improve the accuracy of the effect value (Curtis et al., 1998). The calculation formula of the weighted response ratio and its weight is as follows:


(3) $$RR++=\frac{\sum_{i=1}^{m}\sum_{j=1}^{k}w_{ij}RR_{ij}}{\sum_{i=1}^{m}\sum_{j=1}^{k}w_{ij}}$$

(4) $$w_{ij}=\frac{1}{v}$$

Among them, m is the number of comparison groups, and k is the comparison number of corresponding groups, and $w_{ij}$ is the weight of the effect value of the item ij. The smaller the variance in the study, the greater the weight, indicating that the index is more important in the comprehensive evaluation process. For the convenience of explanation, all the analysis results in this study were expressed as the percentage change of the weighted response ratio RR + +=e^RR++^-1×100%25 (Du et al., 2018).

The standard error of the weighted reaction ratio RR + + is calculated as follows:


(5) $$S(RR++)=\sqrt{\frac{1}{\sum_{i=1}^{m}\sum_{j=1}^{k}w_{ij}}}$$

The 95% confidence interval (95% CI) was calculated as follows:


(6) 95%CI=RR++±1.96×S(RR++)

To ascertain the impact of reduced fertilizer application and water conservation on kiwifruit yield, quality, and water use efficiency (WUE), a meta-analysis was conducted utilizing R - 4.1.0 software. This analysis aimed to compute the average effect size and the corresponding 95% confidence interval. An effect is deemed significant if the 95% confidence interval does not overlap with the line of no effect. Heterogeneity among studies is suggested by a *p*-value greater than 0.1 in conjunction with an I² statistic exceeding 50%, necessitating the use of a random effects model; otherwise, a fixed effects model is appropriate. The meta-analytic outcomes for kiwifruit yield, quality, and WUE, as generated by R, indicated substantial heterogeneity across studies (*p* > 0.1 and I² > 50%), thereby justifying the application of a random effects model. The presence of reporting bias could potentially skew the results of a meta-analysis, with publication bias being particularly influential (Rosenberg, 2005). Egger’s test is a widely recognized quantitative technique for assessing funnel plot asymmetry, serving to identify the presence of publication bias (Egger et al., 1997). The results of this study for kiwifruit yield (fertilization: *p* = 0.36; irrigation: *p* = 0.07), WUE (*p* = 0.24), single fruit weight (*p* = 0.08), soluble sugar (*p* = 0.73), titratable acid (*p* = 0.42), soluble solids (*p* = 0.83), and vitamin C (VC) content (*p* = 0.31) suggested the absence of publication bias. Heterogeneity among studies was evaluated using the I² statistic and Cochran’s Q-test. When I² > 50% and p > 0.1, the heterogeneity was considered substantial, and a random-effects model was applied. Otherwise, a fixed-effects model was used. Egger’s regression test was applied to assess publication bias, with a p-value greater than 0.1 indicating low likelihood of bias (Egger et al., 1997). Additionally, effect sizes were weighted using inverse-variance weighting, which gives greater weight to studies with smaller standard errors and thus higher precision.

## 3 Results

### 3.1 Overview of the dataset

**Figure 3:**
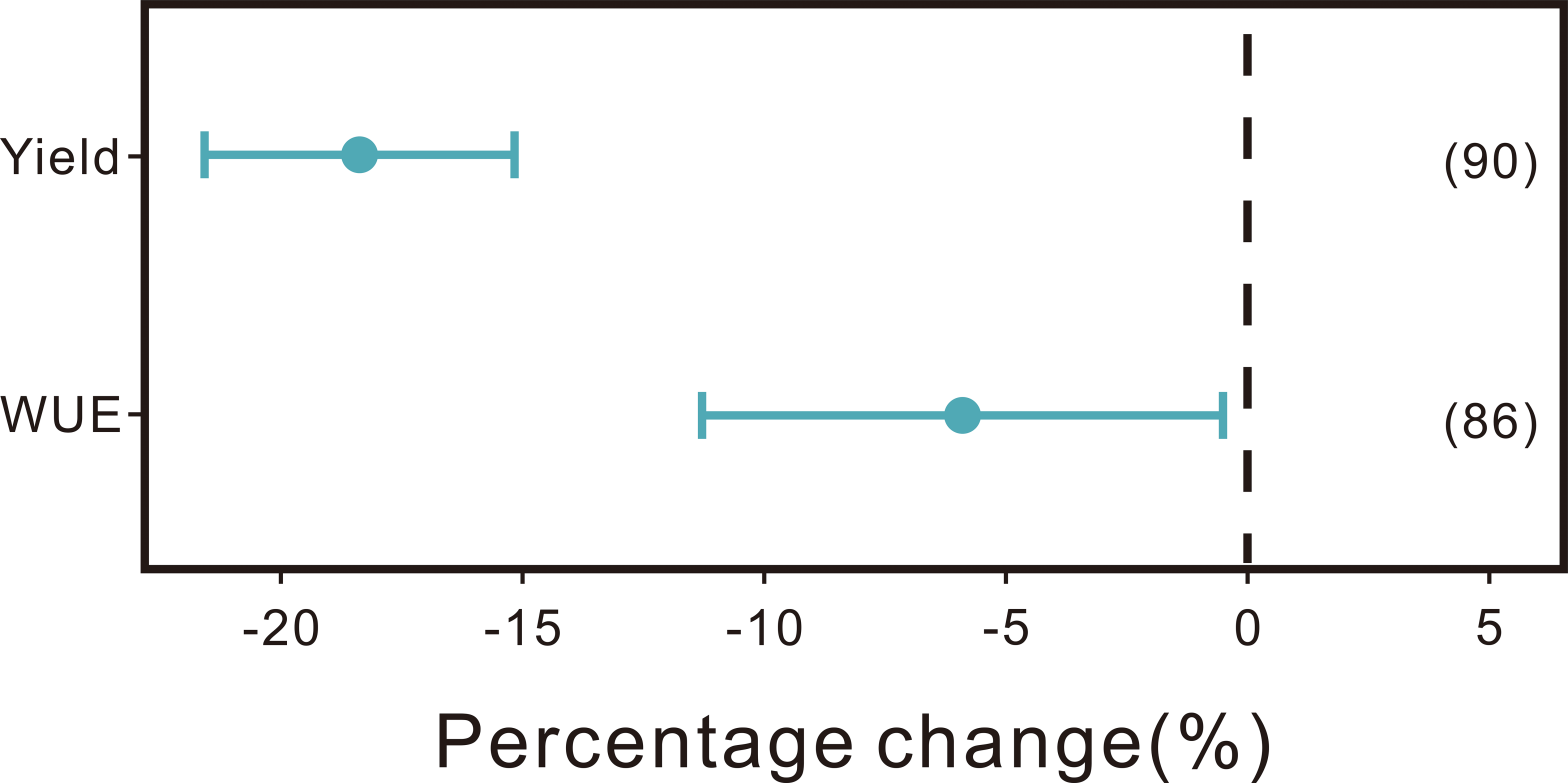
Comparison of the effects of non-optimal water input on kiwifruit yield and water use efficiency (WUE) relative to optimal water input levels. The point estimates and bar plots correspond to the mean effect size and the 95% confidence intervals, respectively. The figures encapsulated in parentheses indicate the number of comparative analyses incorporated within the meta-analytic framework.

**Figure 4:**
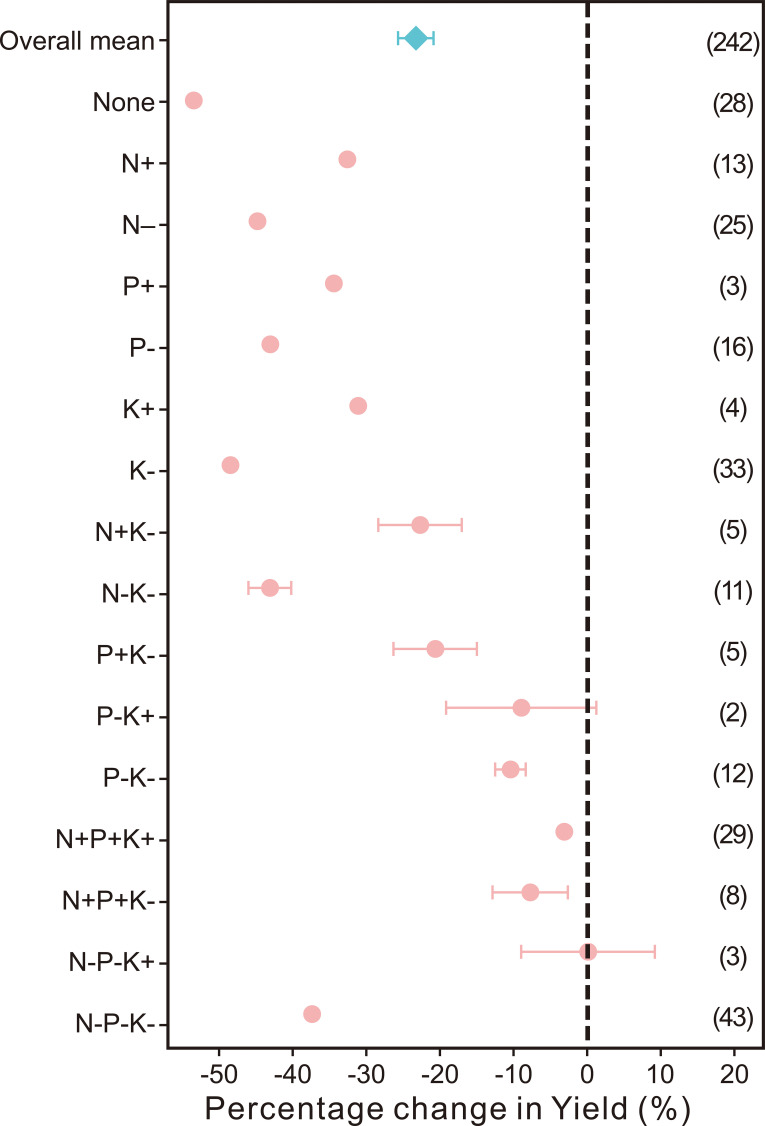
Comparison of the effects of non-optimal fertilization rates on kiwifruit yield relative to optimal fertilization rates. Points and bars represent the average effect and 95% confidence interval, respectively. The numbers in parentheses represent the number of comparisons used in the meta-analysis.

**Figure 5:**
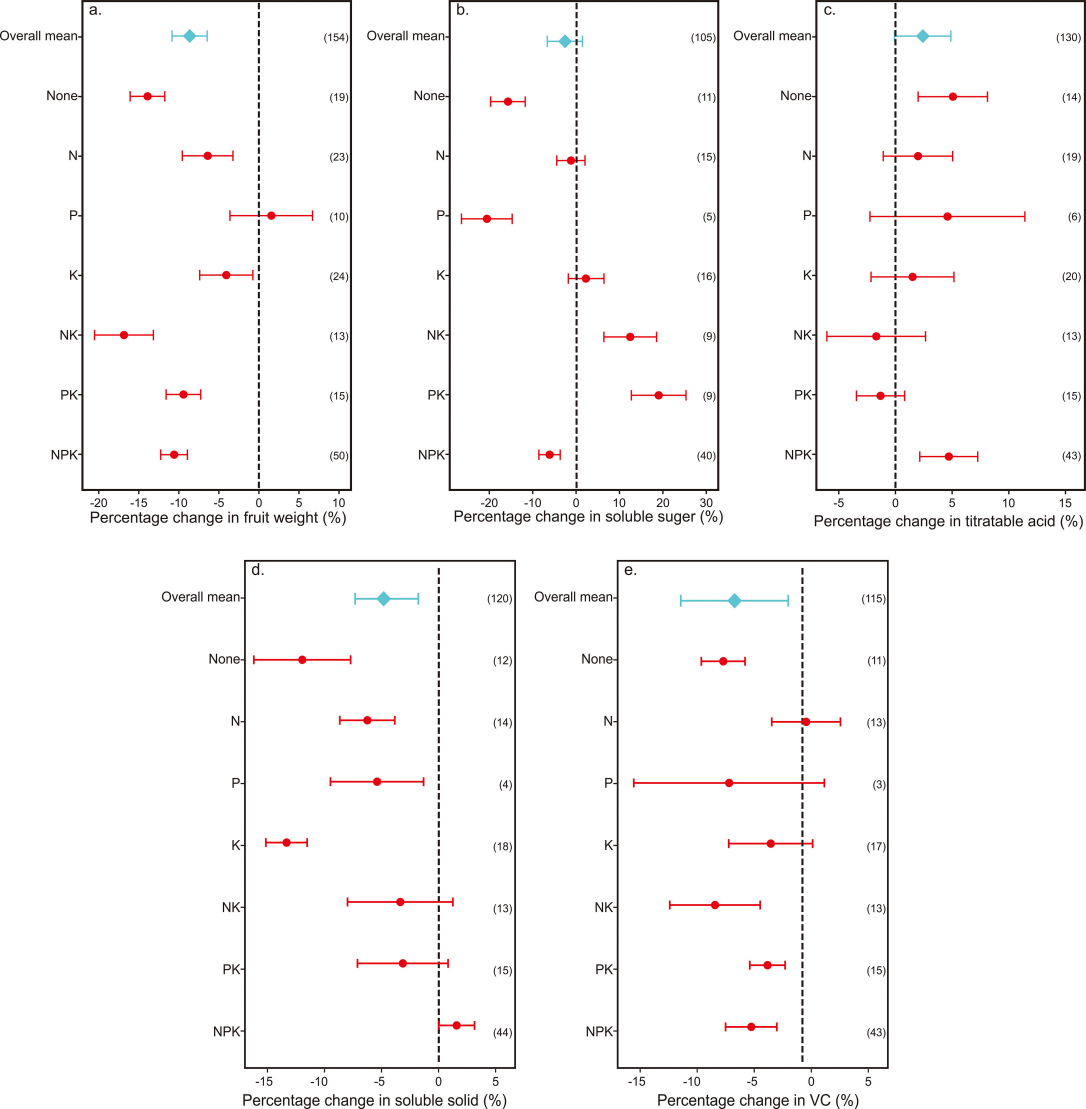
Comparison of the effects of non-optimal fertilization rate on different fruit quality indexes relative to the optimal fertilization rate in kiwifruit. Effects of fertilizer treatments on fruit quality parameters: **(a)** fruit weight; **(b)** soluble sugar; **(c)** titratable acid; **(d)** soluble solids; **(e)** vitamin C. The point and bar plots represent the average effect and the 95% confidence interval, respectively. The numbers in parentheses represent the number of comparisons used in the meta-analysis.

Variations in soil conditions, cultivar selection, and agricultural practices significantly influence the yield, quality, and water use efficiency (WUE) of kiwifruit. On average, non-optimal water input, as compared to the optimal level, has been observed to reduce kiwifruit yield and WUE by 18.18% (95%CI: -21.20% to -15.05%) and 6.03% (95% CI: -11.23% to -0.53%) (**Figure 3**). The aggregate detrimental effects of sub-optimal fertilization on kiwifruit yield, single fruit weight, soluble sugar content, soluble solids, and vitamin C (VC) content were reductions of 23.04% (95% CI: -25.52% to -20.48%), 8.67% (95% CI: -10.82% to -6.46%), 2.74% (95% CI: -6.80% to 1.50%), 4.24% (95% CI: -6.78% to -1.63%), and 6.25% (95% CI: -10.93% to -1.32%), respectively. In contrast, the titratable acidity increased by 2.38% (95% CI: -0.08% to -4.90%) with non-optimal fertilization compared to the optimal condition (**Figures 4**, **5**).

### 3.2 Response of kiwifruit yield and WUE to water and fertilizer input

The impact of sub-optimal fertilizer inputs on kiwifruit yield was examined, revealing significant reductions in yield compared to optimal levels. Specifically, the effects of low phosphorus and potassium (P-K+) and low nitrogen with high potassium (N-P-K+) on yield reduction were not significant. In contrast, non-optimal nitrogen, phosphorus, and potassium fertilizer inputs collectively led to a substantial decrease in kiwifruit yield (**Figure 4**). The most pronounced impact was observed with the absence of fertilization (-53.45%), followed by low potassium (K-) input (-48.27%), and low nitrogen (N-) input (-44.82%). The least effect on yield was attributed to the combination of low nitrogen, low phosphorus, and low potassium (N-P-K+) input (-0.15%), with the next highest effects being high nitrogen, high phosphorus, and high potassium (N+P+K+) input (-3.45%), and high nitrogen, high phosphorus, and low potassium (N+P+K-) input (-7.66%). It is important to note that the effect of the N-P-K+ combination should be further verified due to the small sample size (n=3). When nitrogen, phosphorus, and potassium were applied sub-optimally (N-, P-, K-), the rate of yield reduction was significantly higher than when applied super-optimally (N+, P+, K+). Furthermore, the mixed application of nutrients showed that the effect on yield from high nitrogen and low potassium (N+K-) input (-22.82%) was similar to that of high phosphorus and low potassium (P+K-) input (-20.88%), yet both were substantially less than the effects from low nitrogen and low potassium (N-K-) input (-42.94%) and from the combination of low nitrogen, low phosphorus, and low potassium (N-P-K-) input (-37.38%).

**Figure 6:**
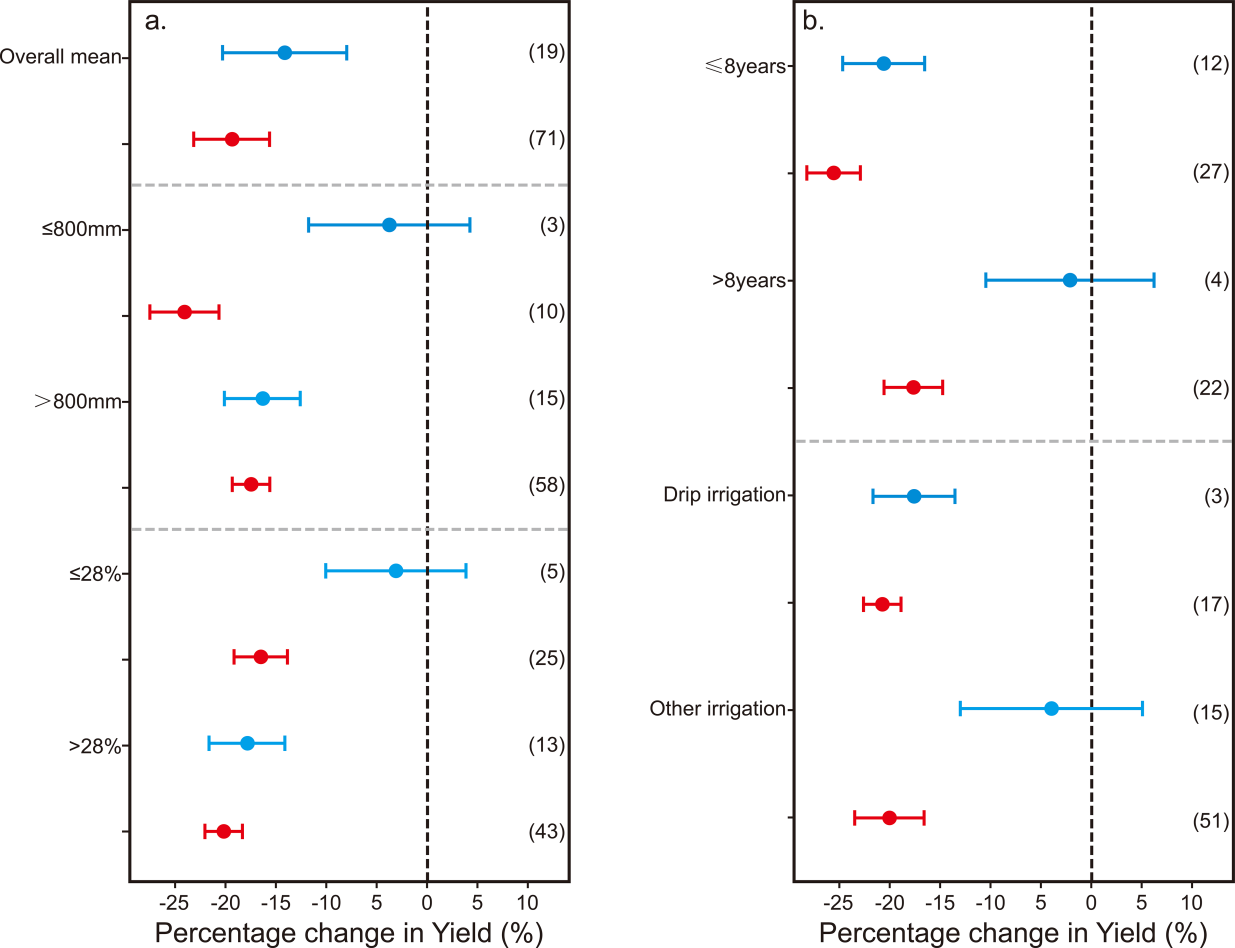
Percentage change in yield across conditions: **(a)** rainfall and slope; **(b)** orchard age and irrigation method.

**Figure 7:**
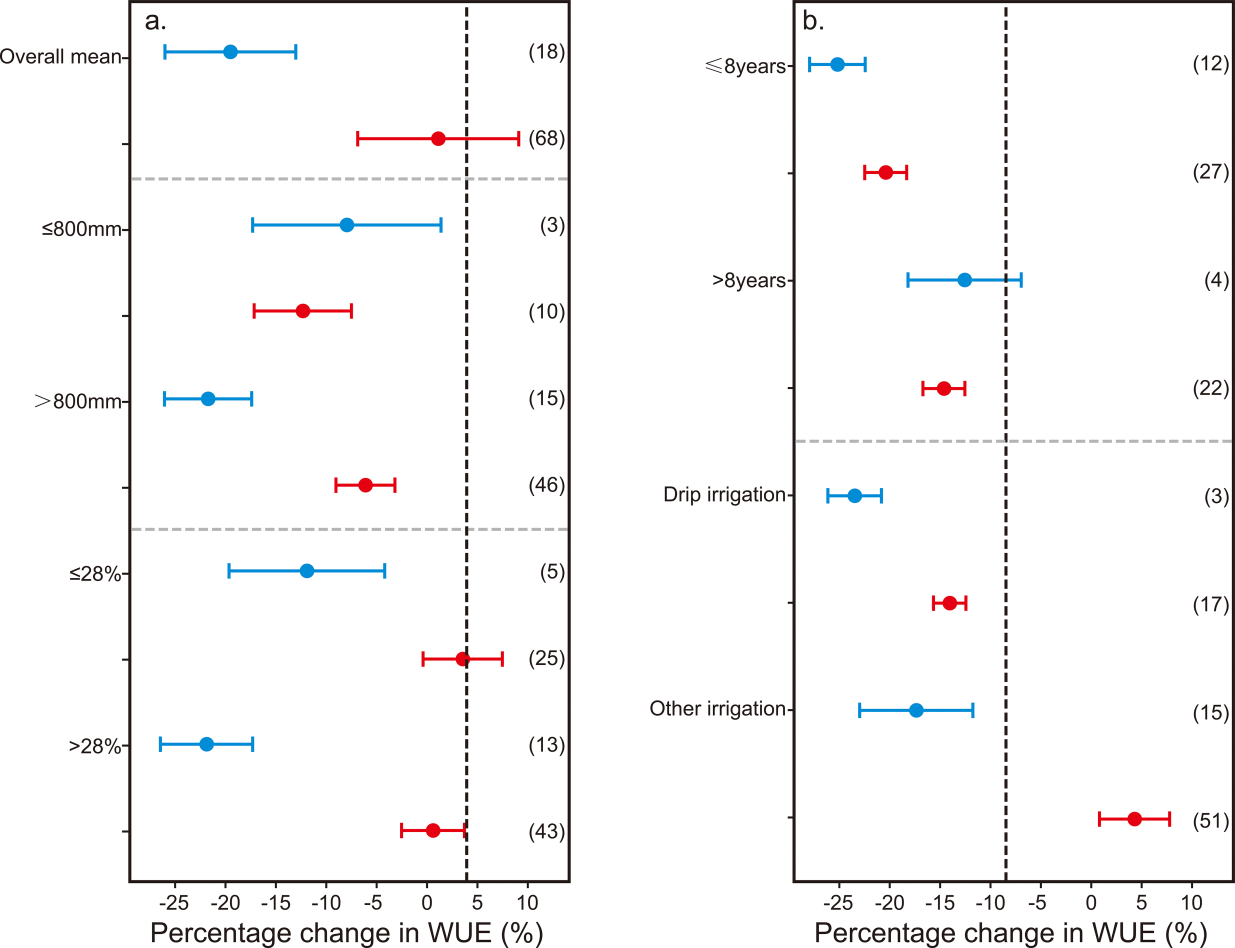
Comparison of the effect of non-optimal water input on the water use efficiency (WUE) of kiwifruit relative to the optimal water input under different annual average rainfall (≤ 800 mm and > 800 mm) **(a)**, field water holding capacity (≤ 28% and > 28%) **(a)**, tree age (≤ 8years and > 8 years) **(b)** and irrigation methods (drip irrigation and other irrigation) **(b)**. The effect of non-optimal water input on the WUE of kiwifruit. Blue (•) indicates SOI water input, and orange (•) indicates sub-optimal water input. The point and bar plots represent the average effect and the 95% confidence interval, respectively. The numbers in parentheses represent the number of comparisons used in the meta-analysis.

The impact of non-optimal water input on kiwifruit yield and WUE was found to be influenced by the annual average rainfall and the field’s water holding capacity (**Figures 6a**, **7a**). Irrespective of these factors, it was observed that super-optimal water input(W+) led to a significant reduction in both kiwifruit yield (by 14.19%) and WUE (by 18.48%). Conversely, sub-optimal water input (W-) resulted in a yield decrease of 18.52%, with its effect on WUE being negligible. When the annual average precipitation exceeded 800mm, the negative impact of super-optimal water input (W+) on kiwifruit yield and WUE was more pronounced, at 16.43% and 20.78%, respectively. In contrast, under conditions of sub-optimal water input (W-) and lower annual average precipitation (≤ 800 mm), the yield and WUE of kiwifruit decreased by 24.12% and 13.06%, respectively. Additionally, under the same field capacity, the sub-optimal water input (W-) had a more substantial effect on kiwifruit yield across different water holding capacities (≤ 28%, by 16.54%; > 28%, by 20.28%), whereas its effect on WUE remained minimal and statistically insignificant.

The influence of non-optimal water input on kiwifruit yield and WUE is modulated by tree age and irrigation techniques (**Figures 6b**, **7b**). The impact of both SOI and sub-optimal water input on kiwifruit yield and WUE attenuates with an increase in tree age. Specifically, SOI water input markedly diminished the yield and WUE of kiwifruit in trees less than 8 years old by 19% and 22.32%, respectively. In contrast, this level of water input did not exert a significant negative effect on trees older than 8 years. Drip irrigation, when applied with SOI water, had a more pronounced negative impact on kiwifruit yield (-16.24%) and WUE (-20.06%) than other irrigation methods. Conversely, under sub-optimal water input, while other irrigation methods could significantly enhance kiwifruit WUE by 17.10%, they generally led to a reduction in yield and WUE. It is noteworthy that under the same irrigation method, the detrimental effect of insufficient irrigation on kiwifruit yield was more pronounced than that of over-irrigation, with the effect on WUE being inversely related.

### 3.3 Response of kiwifruit quality to nitrogen, phosphorus and potassium fertilizer input

#### 3.3.1 Response of single fruit weight of kiwifruit to changes in nitrogen, phosphorus and potassium fertilizers

Except for a sole increase in phosphate fertilizer application, which marginally elevates the individual fruit weight of kiwifruit, alterations in other fertilizer combinations tend to result in a significant decrease in single fruit weight (**Figure 5a**). When juxtaposed with the optimal rate of fertilizer application, it is the variation in nitrogen fertilizer input (NK) that exerts the most substantial impact on kiwifruit’s individual fruit weight, with a reduction of 16.91%. This effect notably surpasses the influence of phosphorus fertilizer input adjustments (PK), which results in a 9.44% decrease. Furthermore, the modulation of nitrogen fertilizer input (N) alone, either increased or decreased, has a slightly more pronounced effect on the single fruit weight of kiwifruit, at a 6.44% reduction, compared to the potassium fertilizer input (K), which yields a 4.12% reduction. The combined application of nitrogen, phosphorus, and potassium fertilizers generally has a more substantial effect on the single fruit weight of kiwifruit than does the application of a single type of fertilizer.

#### 3.3.2 Response of soluble sugar in kiwifruit to changes in nitrogen, phosphorus and potassium fertilizers

The soluble sugar content in kiwifruit was found to be significantly diminished by 6.35% due to the application of SOI levels of nitrogen, phosphorus, and potassium fertilizers. Specifically, alterations in the phosphate fertilizer input (P) exerted the most pronounced effect on kiwifruit’s soluble sugar levels, causing a reduction of 20.59% (**Figure 5b**). However, given the small sample size of n=5, these results warrant further verification. Additionally, the absence of fertilizer treatment had a notably negative impact, decreasing the soluble sugar content by 15.83%. While adjustments to potassium fertilizer input (K) slightly elevated the soluble sugar content of kiwifruit, this effect was not statistically significant. In contrast, when nitrogen or phosphorus fertilizer inputs were modified in conjunction with a reduced potassium fertilizer application, the soluble sugar content of kiwifruit experienced a significant increase of 12.44% for the nitrogen and potassium combination (NK) and 18.87% for the phosphorus and potassium combination (PK), respectively. Furthermore, changes in the combined nitrogen, phosphorus, and potassium (NPK) fertilizer input led to a significant reduction in kiwifruit’s soluble sugar content by 6.1%.

#### 3.3.3 Response of kiwifruit titratable acid to changes of nitrogen, phosphorus and potassium fertilizers

Among the various fertilization treatments, the sole exception to an increase in titratable acidity of kiwifruit was observed when potassium fertilizer was diminished alongside alterations in nitrogen (NK) or phosphorus (PK) fertilizer inputs, resulting in a minor decrease. Conversely, the remaining treatments were associated with an increase in titratable acidity (**Figure 5c**). In contrast to the optimal fertilization rate, both the absence of fertilization and the application of an NPK fertilizer mixture notably elevated the titratable acidity levels in kiwifruit, with comparable variation ranges (No Fertilization: 5.00%; NPK: 4.68%). Adjusting solely the input levels of nitrogen, phosphorus, or potassium fertilizers marginally affects the titratable acidity of kiwifruit, yet the impact is statistically insignificant. The application of fertilizers in quantities exceeding the optimal levels for nitrogen, phosphorus, and potassium notably augmented the titratable acidity of kiwifruit by 4.35%.

#### 3.3.4 Response of soluble solids of kiwifruit to changes in nitrogen, phosphorus and potassium fertilizers

Among the various fertilizer inputs, the alteration in potassium fertilizer input had the most pronounced impact on the soluble solids content of kiwifruit. Adjustments to potassium fertilizer input, whether increases or decreases, resulted in a 12.49% reduction in the percentage change of kiwifruit’s soluble solids (**Figure 5d**). Similarly, no fertilization treatment also significantly reduced the soluble solids content of kiwifruit by 11.25%. The effect of changing the input of nitrogen fertilizer or phosphorus fertilizer on the soluble solids of kiwifruit was similar when the potassium fertilizer was reduced, which decreased by 3.24% (NK) and 2.99% (PK), respectively. Among all fertilization treatments, solely the variation in the NPK fertilizer mixture input demonstrated a positive, albeit minor and statistically insignificant, impact on the soluble solids of kiwifruit. Conversely, the application of fertilizers at levels exceeding the optimal rates for nitrogen, phosphorus, and potassium notably elevated the soluble solids content of kiwifruit by 6.18%.

#### 3.3.5 Response of VC content in kiwifruit to changes in nitrogen, phosphorus and potassium fertilizers

The input of SOI NPK fertilizer significantly reduced the VC content of kiwifruit by 18.37%. Compared with only changing the amount of nitrogen fertilizer input, there was almost no effect on the VC content of kiwifruit; however, various fertilization treatments resulted in a reduction of VC content in kiwifruit (**Figure 5e**). The variation range of VC content in kiwifruit was similar without fertilization and only changing the input of phosphate fertilizer (None: -7.25%; p: -7.06%), but the sample size of the treatment only changing the input of phosphate fertilizer was small (n = 3), and thus the results need to be further verified. Compared with the optimal amount of fertilizer, increasing or decreasing the amount of potassium fertilizer input (K) had little effect on the VC content of kiwifruit (-2.97%), but changing the amount of nitrogen fertilizer input (NK) when the amount of potassium fertilizer decreased would reduce the VC content of kiwifruit by 8.08%, which was higher than the effect of changing the amount of phosphorus fertilizer input (PK) on the VC content of kiwifruit when the amount of potassium fertilizer decreased (-3.23%).

## 4 Discussion

Based on existing literature, this study conducted a systematic and quantitative analysis of the impact of water conservation and fertilizer reduction on the yield and quality of kiwifruit. Based on the meta-analysis of this study, it was found that inappropriate water and fertilizer management significantly reduced kiwifruit yield and water use efficiency (WUE).The meta-analysis revealed that suboptimal fertilization practices led to a decrease in kiwifruit yield, individual fruit weight, soluble sugar content, soluble solids, and vitamin C content by 23.04%, 8.67%, 2.74%, 4.24%, and 6.25%, respectively, while the titratable acidity increased by 2.38% (**Figures 4**, **5**). This outcome is primarily attributed to the fact that excessive fertilization raises the soil’s hydrogen ion concentration, causing soil acidification by decreasing pH (Cui et al., 2013). This, in turn, hinders the complete absorption and utilization of nutrients such as nitrogen, phosphorus, and potassium by plants, thereby adversely affecting the yield and quality of kiwifruit. Furthermore, nitrogen, phosphorus, and potassium are essential nutrients required for plant growth and development (Ahammed et al., 2022). An inadequate supply of nitrogen, phosphorus, and potassium fertilizers can restrict the growth and development of kiwifruit plants, consequently diminishing their yield and quality (Chu et al., 2021).

Reducing the application of SOI levels of nitrogen fertilizer and combined nitrogen, phosphorus, and potassium fertilizers to optimal levels can enhance kiwifruit yield by 32.76% and 3.45%, respectively (**Figure 4**). Studies have indicated that nitrogen application in excess of what is supported by soil, climate, and field management practices may result in yield losses (Zhang et al., 2015; Gao et al., 2012). The detrimental effect of insufficient application of individual nitrogen, phosphorus, and potassium nutrients on kiwifruit yield was found to be significantly greater than that of over-fertilization (**Figure 4**). This can be attributed to the high demand for nitrogen, phosphorus, potassium and other nutrients in kiwifruit. Although this study considered the effects of annual average rainfall and soil water holding capacity on kiwifruit yield and WUE, it may not fully consider the combined effects of other environmental factors (such as temperature, light, etc.). comparative analysis of the yield reduction rates associated with varying inputs of nitrogen, phosphorus, and potassium, particularly under conditions of low nitrogen and potassium (N-K-), revealed a significant decrease in kiwifruit yield by 42.94%. This suggests that kiwifruit has a relatively higher requirement for nitrogen and potassium, while the demand for phosphorus is comparatively lower, aligning with existing research findings. The disproportionate yield response to nitrogen alone *vs*. NPK combinations may be due to nutrient antagonism or dilution effects. Nitrogen promotes vegetative growth, which directly enhances yield, while excess phosphorus and potassium may interfere with nitrogen uptake or balance, reducing synergistic effects (Sardans and Peñuelas, 2021; Liu, 2021). This highlights kiwifruit’s higher demand for N and K compared to P. In correlation with yield results, both excessive and insufficient applications of nitrogen and potassium fertilizers were found to significantly reduce the single fruit weight of kiwifruit (**Figure 5a**).

Conversely, adjusting the application amount of phosphate fertilizer increased the single fruit weight of kiwifruit by 1.32% compared to the optimal fertilization level, potentially due to variations in the number of fruits per plant among the sampled kiwifruit trees. The impact of combined nitrogen, phosphorus, and potassium fertilizer inputs on the single fruit weight of kiwifruit was generally more pronounced than that of single nutrient applications. This synergistic effect is attributed to the interactive relationships between these three essential nutrient elements (Chu et al., 2021). Potassium notably enhances the absorption and utilization efficiency of nitrogen by plants, facilitating its rapid conversion into protein, which in turn, contributes to an increase in the single fruit weight of kiwifruit (Liu, 2021)Although the effect of nitrogen (−16.91%) and phosphorus (−9.44%) fertilizer inputs on single fruit weight appeared notably different, the confidence intervals of these estimates partially overlap, indicating that the statistical significance of the difference between them may be limited. Further subgroup analyses by environmental factors such as tree age, rainfall, and soil capacity were constrained by limited sample size in the included studies. Nonetheless, the more pronounced effect of nitrogen may reflect its direct role in cell division and expansion during fruit development (Liu, 2021), whereas phosphorus primarily affects energy metabolism. These physiological differences might explain the observed magnitude gap.

Kiwifruit, known for its nutritional richness, exhibits varying responses in nutrient content to different fertilizer application rates. This study analyzed the impact of fertilization on kiwifruit quality, ensuring conditions were optimal for maximal yield. Relative to the optimal fertilization strategy, modifications in nitrogen fertilizer levels had minimal influence on the levels of soluble sugars, titratable acidity, and vitamin C (VC) in kiwifruit (**Figures 5b, c, e**). This insignificance may stem from the specific nitrogen requirements of kiwifruit, where an appropriate nitrogen input can notably enhance individual fruit weight without compromising fruit quality. Conversely, a reduction in soluble sugar, soluble solids, and VC content, and a rise in titratable acidity were observed in kiwifruit (**Figures 5b–e**). Phosphorus, integral to plant nutrition, is a constituent of key compounds including nucleic acids, proteins, and enzymes. Insufficient phosphorus can restrict plant growth, development, and metabolism (Poirier and Bucher, 2002; Sardans and Peñuelas, 2021). This study revealed that adjusting the levels of nitrogen, phosphorus, and potassium fertilizers to match the application rates at the peak yield point could markedly elevate the soluble sugar and VC content in kiwifruit by 6.35% and 18.37%(**Figures 5b, e**). Conversely, the titratable acidity and soluble solids content were significantly lowered by 4.35% and 6.18%(**Figures 5c, d**). The role of potassium in plant cells includes osmotic regulation and maintaining ionic balance. An excessive application of potassium fertilizer may lead to competition between potassium ions and other ions, such as Ca2+, Mg2+, NH4+, etc., for entry into fruit cells (Srivastava et al., 2020; Sustr et al., 2019; Xu et al., 2020; Panda et al., 2012; Sangha et al., 2023), potentially inhibiting VC synthesis. Furthermore, an overabundance of potassium can escalate the soluble solids content within the fruit, disrupting its acid-base equilibrium and elevating the fruit’s pH value. This shift can accelerate fruit rancidity, thereby impairing fruit quality and flavor.

The yield and WUE of kiwifruit experienced respective increases of 14.19% and 18.48%, when SOI water input was adjusted to optimal levels. Conversely, augmenting sub-optimal water input to optimal levels resulted in yield and WUE increases of 18.52% and 1.57%, respectively (**Figures 6a**, **7a**). These findings underscore the critical importance of precise water management. Over-irrigation may induce root hypoxia in kiwifruit and contribute to nutrient leaching from the soil, consequently diminishing yield and WUE (Li et al., 2014) Insufficient water supply, on the other hand, causes evident stress symptoms in plants, impeding normal growth and development (Yang et al., 2012; Pahalvi et al., 2021).

Subgroup analysis revealed that the impact of non-optimal water input on kiwifruit yield and WUE is contingent upon annual average rainfall and the field’s water holding capacity (**Figures 6a**, **7a**). In regions with an annual average rainfall exceeding 800 mm and a field water holding capacity above 28%, the adverse effects of SOI water input on kiwifruit yield and WUE were markedly more pronounced than in areas with lower rainfall and water holding capacity. This disparity may arise from the enhanced water holding capacity of soils in regions with higher precipitation and water holding capacity. Excessive irrigation in such areas can lead to waterlogging and surface runoff, surpassing the water requirements of kiwifruit plants and thus reducing their WUE (Yang et al., 2012). Over-saturation of soil can impede oxygen circulation, degrade soil aeration, curtail microbial populations and activity, and adversely affect soil fertility and health (Wei et al., 2021).

Moreover, in low-lying areas with poor drainage, soil saturation can exacerbate salinization and potentially result in root death and decline of kiwifruit plants. The influence of non-optimal water input on kiwifruit yield and WUE is also moderated by tree age. The study confirmed that the negative effects of both SOI and sub-optimal water input on yield and WUE are mitigated with increasing tree age (**Figures 6b**, **7b**). This outcome can be ascribed to the more extensive root systems of mature kiwifruit trees, which foster a more stable ecosystem, bolster environmental and soil adaptability, and confer greater resilience to drought and flood, thereby decreasing reliance on irrigation water.

Additionally, as kiwifruit trees age, their leaf area expands, escalating transpiration rates and influencing WUE. Appropriate irrigation methods are pivotal for enhancing crop yields. Meta-analysis results indicated that under drip irrigation, reducing SOI water input to optimal levels significantly increased kiwifruit yield and WUE by 16.24% and 20.06%, respectively—outperforming other irrigation techniques (**Figures 6b**, **7b**). This superiority is largely attributed to the distinct soil water and salt transport characteristics associated with different irrigation methods (Yu et al., 2020). Drip irrigation delivers water directly to the root zone, enabling precise soil moisture management while minimizing surface evaporation and the risk of soil salinization. In contrast, other irrigation methods, such as tubular outflow, may lead to water pooling and localized hypoxia, which can significantly impede root respiration and the transport of water and nutrients. Nonetheless, other irrigation methods have been observed to enhance kiwifruit WUE under sub-optimal water input conditions (**Figure 7b**), possibly due to variations in irrigation water application across studies. Furthermore, micro-sprinkler irrigation and combined drip-sprinkler systems can more effectively manage water distribution, reducing deep percolation losses.

## 5 Conclusions

This paper presents a meta-analysis of field experimental data from kiwifruit production across various growth environments in China. This study demonstrates that optimizing nitrogen, phosphorus, and potassium (NPK) fertilizer levels, along with water input, can significantly improve kiwifruit yield and water use efficiency (WUE). In particular, reducing nitrogen fertilization to optimal levels enhances yield and quality, highlighting kiwifruit’s higher demand for nitrogen and potassium compared to phosphorus. These findings provide valuable insights into water and fertilizer management for kiwifruit cultivation. These findings indicate that kiwifruit exhibits a relatively higher demand for nitrogen and potassium compared to phosphorus. Furthermore, by optimizing water input, both the yield and WUE of kiwifruit were increased by 14.19% and 18.48%, respectively. In regions with an annual average rainfall exceeding 800 mm and a field water holding capacity above 28%, the detrimental effects of SOI water input on kiwifruit yield and WUE were considerably more pronounced than in regions with lower rainfall and water holding capacity. The impact of both SOI and sub-optimal water input on kiwifruit yield and WUE was found to diminish with the increase in tree age. Drip irrigation, in particular, emerged as a superior water-saving technique in kiwifruit orchards, significantly boosting yield and WUE by 16.24% and 20.06%, respectively. Based on the results of this study, future research can further explore the changes in the response of kiwifruit to water and fertilizer reduction at different growth stages, and the best water and fertilizer management strategies under different climatic and soil conditions. In addition, the inclusion of economic cost analysis will help farmers and decision-makers to better balance resource utilization and economic benefits in practical applications. Future studies should integrate temperature and light conditions to further refine irrigation and fertilization recommendations tailored to local environments. In addition, these results highlight the importance of defining region-specific thresholds for water and nutrient inputs, as environmental factors such as rainfall and soil capacity vary widely across kiwifruit-producing areas. Future research should also incorporate economic input–output analysis, soil microbial community responses, and long-term sustainability modeling to develop more integrated and practical irrigation-fertilization strategies for farmers. Future studies should integrate temperature and light conditions to further refine irrigation and fertilization recommendations tailored to local environments.

## Data Availability

The original contributions presented in the study are included in the article/**Supplementary Material**. Further inquiries can be directed to the corresponding authors.

## References

[B1] Ahammed G. J. Chen Y. Liu C. Yang Y. (2022). Light regulation of potassium in plants. Plant Physiol. Biochem. 170, 316–324. doi: 10.1016/j.plaphy.2021.12.019, PMID: 34954566

[B2] Busschaert L. De Roos S. Thiery W. Raes D. De Lannoy G. J. M. (2022). Net irrigation requirement under different climate scenarios using AquaCrop over Europe. Hydrol. Earth Syst. Sci. Discuss. 2022, 1–30. doi: 10.5194/hess-26-3731-2022

[B3] Chu C. Wang Y. Wang E. (2021). Research status and prospect of efficient utilization of nitrogen, phosphorus, and potassium in plants. Chin. Sci.: Life Sci. 51, 1415–1423. doi: 10.1360/SSV-2021-0163

[B4] Cui S. H. Shi Y. L. Groffman P. M. Schlesinger W. H. Zhu Y. G. (2013). Centennial-scale analysis of the creation and fate of reactive nitrogen in China, (1910 - 2010). Proc. Natl. Acad. Sci. 110, 2052–2057. doi: 10.1073/pnas.1221638110, PMID: 23341613 PMC3568337

[B5] Cui K. Shoemaker S. P. (2018). A look at food security in China. NPJ Sci. Food 2, 4. doi: 10.1038/s41538-017-0016-2, PMID: 31304254 PMC6550196

[B6] Curtis P. S. Wang A. (1998). A meta-analysis of elevated CO2 effects on woody plant mass, form, and physiology. Oecologia 113, 299–313. doi: 10.1007/s004420050381, PMID: 28307814

[B7] Dai Z. G. Fei L. J. Huang D. L. Zeng J. Chen L. Cai Y. H. (2019). Coupling effects of irrigation and nitrogen levels on yield, water and nitrogen use efficiency of surge-root irrigated jujube in a semiarid region. Agric. Water Manage. 213, 146–154. doi: 10.1016/j.agwat.2018.09.035

[B8] Davidson E. A. Suddick E. C. Rice C. W. Prokopy L. S. (2015). More food, low pollution (Mo Fo Lo Po): a grand challenge for the 21st century. J. Environ. Qual. 44, 305–311. doi: 10.2134/jeq2014.09.0340, PMID: 26023950

[B9] Du Y. D. Niu W. Q. Gu X. B. Zhang Q. Cui B. J. Zhao Y. (2018). Crop yield and water use efficiency under aerated irrigation: A meta-analysis. Agric. Water Manage. 210, 158–164. doi: 10.1016/j.agwat.2018.07.038

[B10] Edwards J. D. Pittelkow C. M. Kent A. D. Yang W. H. (2018). Dynamic biochar effects on soil nitrous oxide emissions and underlying microbial processes during the maize growing season. Soil Biol. Biochem. 122, 81–90. doi: 10.1016/j.soilbio.2017.12.017

[B11] Egger M. Smith G. D. Phillips B. A. &sidious S. (1997). Bias in meta-analysis detected by a simple, graphical test. BMJ 315, 629–634. doi: 10.1136/bmj.315.7109.629, PMID: 9310563 PMC2127453

[B12] Gao Q. Li C. Feng G. Wang J. Cui Z. Chen X. . (2012). Understanding yield response to nitrogen to achieve high yield and high nitrogen use efficiency in rainfed corn. Agron. J. 104, 165–168. doi: 10.2134/agronj2011.0215

[B13] Gao Z. Zhang X. Chen L. Lu Y. Mao J. Wang X. . (2023). Study on the water consumption law of ‘Jinguo’ kiwifruit in hilly gentle slope. Northern. Horticult. 02, 17–24.

[B14] Gong H. Yin Y. Chen Z. Zhang Q. Tian X. Wang Z. . (2025). A dynamic optimization of soil phosphorus status approach could reduce phosphorus fertilizer use by half in China. Nat. Commun. 16, 976. doi: 10.1038/s41467-025-56178-1, PMID: 39856072 PMC11761064

[B15] Gu X. B. Li Y. N. Du Y. D. (2017). Optimized nitrogen fertilizer application improves yield, water and nitrogen use efficiencies of winter rapeseed cultivated under continuous ridges with film mulching. Ind. Crops Products. 109, 233–240. doi: 10.1016/j.indcr.2017.01.017

[B16] Guarçoni M. A. Ventura J. A. (2011). Nitrogen, P, and K fertilization and the development, yield, and fruit quality of pineapple ‘gold’ (MD - 2). Revista Brasileira de Ciência do Solo Uncited. 35(4), 1367–1376. doi: 10.1590/S0100-06832011000400031

[B17] Hawkesford M. J. (2014). Reducing the reliance on nitrogen fertilizer for wheat production. J. Cereal Sci. 59, 276–283. doi: 10.1016/j.jcs.2014.05.005, PMID: 24882935 PMC4026125

[B18] Hedges L. V. Gurevitch J. Curtis P. S. (1999). The meta-analysis of response ratios in experimental ecology. Ecology 80, 1150–1156. doi: 10.1890/0012-9658(1999)080[1150:TMAORR]2.0.CO;2

[B19] Khapte P. S. Kumar P. Burman U. Kumar P. (2019). Deficit irrigation in tomato: Agronomical and physio-biochemical implications. Sci. Hortic. 248, 256–264. doi: 10.1016/j.scienta.2019.01.006

[B20] Kuypers M. M. Marchant H. K. Kartal B. (2018). The microbial nitrogen-cycling network. Nat. Rev. Microbiol. 16, 263–276. doi: 10.1038/s41579-018-0046-0, PMID: 29398704

[B21] Li Y. (2022). Effects of branch traction on growth, yield and quality of kiwifruit and its mechanism (Ya’an, China: Sichuan Agricultural University). doi: 10.27345/d.cnki.gsnyu.2022.001102

[B22] Li J. Zhang F. C. Fang D. Li Z. Gao M. Wang H. . (2014). Effects of water and nitrogen supply on cucumber growth and water use under drip fertigation. Chin. Agric. Sci. 47, 4475–4487. doi: 10.3864/j.issn.0578-1752.2014.22.013

[B23] Li Y. Li Z. Cui S. Jagadamma S. Zhang Q. P. (2019). Residue retention and minimum tillage improve physical environment of the soil in croplands: A global meta-analysis. Soil Tillage. Res. 194, 104292. doi: 10.1016/j.stres.2019.104292

[B24] Li H. R. Mei X. Wang J. Huang F. Hao W. Li B. G. (2021). Drip fertigation significantly increased crop yield, water productivity, and nitrogen use efficiency with respect to traditional irrigation and fertilization practices: A meta-analysis in China. Agric. Water Manage. 244, 106534. doi: 10.1016/j.agwat.2020.106534

[B25] Li Y. M. Sun Y. X. Liao S. Q. Zou G. Y. Zhao T. K. Chen Y. H. . (2017). Effects of two slow-release nitrogen fertilizers and irrigation on yield, quality, and water-fertilizer productivity of greenhouse tomato. Agric. Water Manage. 186, 139–146. doi: 10.1016/j.agwat.2017.08.020

[B26] Li M. F. Wang J. Guo D. Yang R. R. Fu H. (2019). Effect of land management practices on the concentration of dissolved organic matter in soil: A meta-analysis. Geoderma 344, 74–81. doi: 10.1016/j.geoderma.2019.05.024

[B27] Li Z. Zhang Q. Wei W. Cui S. Tang W. Li Y. (2020). Determining effects of water and nitrogen inputs on wheat yield and water productivity and nitrogen use efficiency in China: A quantitative synthesis. Agric. Water Manage. 242, 106397. doi: 10.1016/j.agwat.2020.106397

[B28] Liu D. (2021). Root developmental responses to phosphorus nutrition. J. Integr. Plant Biol. 63, 1065–1090. doi: 10.1111/jipb.13090, PMID: 33710755

[B29] Liu Y. Shi J. Li J. (2000). Miliang No.1, a delicious kiwifruit nutrient element and its seasonal variation. J. Jishou. Univ. 01, 6–10.

[B30] Lu Y. L. Chen Z. J. Kang T. Zhang X. J. Bellarby J. Zhou J. B. (2016). Land-use changes from arable crop to kiwi-orchard increased nutrient surpluses and accumulation in soils. Agricult. Ecosyst. Environ. 223, 270–277. doi: 10.1016/j.agee.2016.04.017

[B31] Luo Y. Q. Hui D. F. Zhang D. Q. (2006). Elevated CO2 stimulates net accumulations of carbon and nitrogen in land ecosystems: A meta-analysis. Ecology 87, 53–63. doi: 10.1890/04-1724, PMID: 16634296

[B32] Lv H. . (2020). Conventional flooding irrigation and over fertilization drives soil pH decrease not only in the top-but also in subsoil layers in solar greenhouse vegetabe production systems. Geoderma 363, 114156. doi: 10.1016/j.geoderma.2020.114156

[B33] Muhammad I. Yang L. Ahmad S. Farooq S. Al-Ghamdi A. A. Khan A. . (2022). Nitrogen fertilizer modulates plant growth, chlorophyll pigments, and enzymatic activities under different irrigation regimes. Agronomy 12, 845. doi: 10.3390/agronomy12040845

[B34] Pacheco C. Souza L. C. Lemos T. L. Pinheiro J. C. Gomes R. C. (2008). Influence of nitrogen and potassium on yield, fruit quality, and mineral composition of kiwifruit. Int. J. Energy Environ. 2, 9–15.

[B35] Pahalvi H. N. . (2021). “Chemical fertilizers and their impact on soil health,” in Ecofriendly tools for reclamation of degraded soil environs, Cham, Switzerland: Springer. vol. 2. , 1–20. Microbiota and Biofertilizers.

[B36] Panda B. B. Sharma S. Mohapatra P. K. Das A. (2012). Application of excess nitrogen, phosphorus, and potassium fertilizers leads to lowering of grain iron content in high-yielding tropical rice. Commun. Soil Sci. Plant Anal. 43, 2590–2602. doi: 10.1080/00103624.2012.716122

[B37] Patanè C. Cosentino S. L. (2009). Effects of soil water deficit on yield and quality of processing tomato under a Mediterranean climate. Agric. Water Manage. 97, 131–138. doi: 10.1016/j.agwat.2009.08.021

[B38] Pinto R. Brito L. M. Rodrigues J. R. O. Rego R. Moura L . (2021). Influence of irrigation and nitrogen fertilization on kiwifruit production. Agric. Eng. 707, 2021–2021. doi: 10.1016/j.ageki.2021.100709

[B39] Poirier Y. Bucher M. (2002). Phosphate transport and homeostasis in Arabidopsis. Arabidopsis. Book. 1, e0024. doi: 10.1199/tab.0024, PMID: 22303200 PMC3243343

[B40] Rosenberg M. S. (2005). The file-drawer problem revisited: a general weighted method for calculating fail-safe numbers in meta-analysis. Evolution 59, 464–468. doi: 10.1111/j.0014-3820.2005.tb01004, PMID: 15807430

[B41] Sangha L. Shortridge J. Frame W. (2023). The impact of nitrogen treatment and short-term weather forecast data in irrigation scheduling of corn and cotton on water and nutrient use efficiency in humid climates. Agric. Water Manage. 283, 108314. doi: 10.1016/j.agwat.2023.108314

[B42] Santoni F. Petruccioli M. Goni F. Daolio G. Dupont J. M. Motta R. (2016). Influence of cultivation parameters on the mineral composition of kiwi fruit from Corsica. Chem. Biodivers. 13, 748–754. doi: 10.1002/cbdv.201500236, PMID: 27135990

[B43] Sardans J. Peñuelas J. (2021). Potassium control of plant functions: Ecological and agricultural implications. Plants 10, 419. doi: 10.3390/plants10020419, PMID: 33672415 PMC7927068

[B44] Satpal D. Kaur J. Bhadariya V. Sharma K. (2021). Actinidia deliciosa (Kiwi fruit): A comprehensive review on the nutritional composition, health benefits, traditional utilization, and commercialization. J. Food Process. Preserv. 45, e15588. doi: 10.1111/jfpp.15588

[B45] Srivastava A. K. Shankar A. Nalini Chandran A. K. Sharma M. Jung K. H. Suprasanna P. . (2020). Emerging concepts of potassium homeostasis in plants. J. Exp. Bot. 71, 608–619. doi: 10.1093/jxb/erz458, PMID: 31624829

[B46] Stanley T. D. (2001). Wheat from chaff: Meta-analysis as quantitative literature review. J. Econ. Perspect. 15, 131–150. doi: 10.1257/jep.15.3.131

[B47] Sustr M. Soukup A. Tylova E. (2019). Potassium in root growth and development. Plants 8, 435. doi: 10.3390/plants8100435, PMID: 31652570 PMC6843428

[B48] Tilman D. Cassman K. G. Matson P. A. Naylor R. L. Polasky S. (2011). Global food demand and the sustainable intensification of agriculture. Proc. Natl. Acad. Sci. 108, 20260–20264. doi: 10.1016/j.croj.2015.05.001 22106295 PMC3250154

[B49] Wang G. Y. Hu Y. X. Liu Y. X. Ahmad S. Zhou X. B. (2021). Effects of supplement irrigation and nitrogen application levels on soil carbon–nitrogen content and yield of one-year double crop maize in subtropical region. Water 13, 1180. doi: 10.3390/w13091180

[B50] Wang L. L. Palta J. A. Chen W. Chen Y. L. Deng X. P. (2018b). Nitrogen fertilization improved water-use efficiency of winter wheat through increasing water use during vegetative rather than grain filling. Agric. Water Manage. 197, 41–53. doi: 10.1016/j.agwat.2018.07.002

[B51] Wang L. F. Sun J. T. Zhang Z. B. Xu P. Shangguan Z. P. (2018a). Winter wheat grain yield in response to different production practices and soil fertility in northern China. Soil Tillage. Res. 176, 10–17. doi: 10.1016/j.still.2017.10.001

[B52] Wang W. Zhuo L. Li M. Liu Y. L. Wu P. T. (2019). The effect of development in water-saving irrigation techniques on spatial-temporal variations in crop water footprint and benchmarking. J. Hydrol. 577, 123916. doi: 10.1016/j.jhydrol.2019.123916

[B53] Wei C. C. Ren S. M. Yang P. L. Wang Y. He X. Xu Z. . (2021). Effects of irrigation methods and salinity on CO2 emissions from farmland soil during growth and fallow periods. Sci. Total. Environ. 752, 141639. doi: 10.1016/j.scitotenv.2020.141639, PMID: 32890824

[B54] Wu J. Song Y. Wan G.-Y. Sun L.-Q. Wang J.-X. Zhang Z.-S. . (2025). Boosting crop yield and nitrogen use efficiency: the hidden power of nitrogen-iron balance. New Crops 2, 100047. doi: 10.1016/j.ncrops.2024.100047

[B55] Wu H. B. Wang L. C. Kang L. A. Liu C. Li M. W. (2023). Study on the effect of planting pattern adjustment on the growth of kiwifruit inter-root microorganisms and fruit quality. Turkish. J. Agric. Forestry. 47, 263–272. doi: 10.17766/tras.200002

[B56] Wu Y. Zhang X. Tang X. Cheng K. Fu C. Chen X. (2021). Responses of growth, fruit yield, quality, and water productivity of greenhouse tomato to deficit drip irrigation. Sci. Hortic. 275, 109710. doi: 10.1016/j.scienta.2020.109710

[B57] Xu X. X. Du X. Wang F. Sha J. C. Chen Q. Tian G. . (2020). Effects of potassium levels on plant growth, accumulation and distribution of carbon, and nitrate metabolism in apple dwarf rootstock seedlings. Front. Plant Sci. 11, 534048. doi: 10.3389/fpls.2020.00904, PMID: 32655607 PMC7325393

[B58] Yang A. . (2017). Optimising nitrogen fertilisation: A key to improving nitrogen-use efficiency and minimising nitrate leaching losses in an intensive wheat/maize rotation, (2008–2014). Field Crops Res. 206, 1–10. doi: 10.1016/j.fcr.2016.12.011

[B59] Yang Q. Zhang F. Liu X. Ge Z. (2012). “Effects of drip irrigation mode and NaCl concentration on hydraulic resistance and water use of young apple tree,” in Transactions of the Chinese Society of Agricultural Engineering, vol. 28. (Editorial Office of Transactions of the Chinese Society of Agricultural Engineering), 117–123.

[B60] Yi J. Li H. Zhao Y. Shao M. Zhang H. (2022). Assessing soil water balance to optimize irrigation schedules of flood-irrigated maize fields with different cultivation histories in the arid region. Agric. Water Manage. 266, 107543. doi: 10.1016/j.agwat.2022.107543

[B61] Yu L. Y. Zhao X. N. Gao X. D. Siddique K. H. M. (2020). Improving/maintaining water-use efficiency and yield of wheat by deficit irrigation: A global meta-analysis. Agric. Water Manage. 228, 105906. doi: 10.1016/j.agwat.2019.105906

[B62] Zhang S. L. Gao P. C. Tong Y. A. Norse D. Lu Y. L. Powlson D. (2015). Overcoming nitrogen fertilizer over-use through technical and advisory approaches: A case study from Shaanxi Province, northwest China. Agricult. Ecosyst. Environ. 209, 89–99. doi: 10.1016/j.agee.2015.03.002

[B63] Zhao Y. Kang Z. Chen L. Guo Y. Mu Q. Wang S. . (2023). Quality classification of kiwifruit under different storage conditions based on deep learning and hyperspectral imaging technology. J. Food Measurement. Characterization. 17, 289–305. doi: 10.1016/j.jfmc.2022.100824

[B64] Zhou J. Y. Gu B. J. Schlesinger W. H. Ju X. T. (2016). Significant accumulation of nitrate in Chinese semi-humid croplands. Sci. Rep. 6, 25088. doi: 10.1038/srep25088, PMID: 27114032 PMC4844977

